# Twelve Novel *Atm* Mutations Identified in Chinese Ataxia Telangiectasia Patients

**DOI:** 10.1007/s12017-013-8240-3

**Published:** 2013-06-27

**Authors:** Yu Huang, Lu Yang, Jianchun Wang, Fan Yang, Ying Xiao, Rongjun Xia, Xianhou Yuan, Mingshan Yan

**Affiliations:** 1 Zijing Biomedical Institute, School of Medicine, Wuhan University of Science and Technology, 820 Heping Street, Wuhan, 430062 China; 2 Department of Medical Genetics, Peking University Health Science Center, Beijing, China; 3 Bach Pharma, Inc., 800 Turnpike Street, Suite 300, North Andover, MA 01845 USA

**Keywords:** Ataxia telangiectasia, Mutation analysis, Sequencing, MLPA

## Abstract

Ataxia telangiectasia (A-T) is an autosomal recessive disease characterized mainly by progressive cerebellar ataxia, oculocutaneous telangiectasia, and immunodeficiency. This disease is caused by mutations of the ataxia telangiectasia mutated (*Atm*) gene. More than 500 *Atm* mutations that are responsible for A-T have been identified so far. However, there have been very few A-T cases reported in China, and only two Chinese A-T patients have undergone *Atm* gene analysis. In order to systemically investigate A-T in China and map their *Atm* mutation spectrum, we recruited eight Chinese A-T patients from six unrelated families nationwide. Using direct sequencing of genomic DNA and the multiplex ligation-dependent probe amplification, we identified twelve pathogenic *Atm* mutations, including one missense, four nonsense, five frameshift, one splicing, and one large genomic deletion. All the *Atm* mutations we identified were novel, and no homozygous mutation and founder-effect mutation were found. These results suggest that *Atm* mutations in Chinese populations are diverse and distinct largely from those in other ethnic areas.

## Introduction

The hallmark of A-T is progressive neurodegeneration, manifested as cerebellar ataxia (Boder et al. 1958; Barlow et al. 1996). A-T occurs in early childhood, with an incidence varying from 1 in 40,000 to 100,000 births in various ethnic areas (Swift et al. 1986), and caused by biallelic mutations of *Atm* gene located on chromosome 11q23.1 (Gatti et al. 1988). *Atm* gene, identified in 1995 (Savitsky et al. 1995), is very large and is comprised of 66 exons with an open reading frame of 9,168 nucleotides. *Atm* gene product, ATM, is a protein kinase with 3,050 amino acids and belongs to the phosphoinositide 3-kinase-related protein kinase super family. ATM is mainly located in the nucleus, although it has been found in cytosol associated with peroxisomes (Watters et al. 1999). As a multifunctional protein kinase, ATM, upon its autophosphorylation, plays a critical role in regulation of cell cycle control, DNA damage and repair, and cell survival and death by orchestrating the phosphorylation of multiple substrates (Goodarzi et al. 2004; Kozlov et al. 2011). As a caretaker, ATM, which also is a redox thiol-sensitive protein kinase, functions by activating multiple redox-sensitive or phosphorylation-sensitive mechanisms responsible for maintaining genomic, telomeric, and chromosomal integrity under conditions of genomic or redox stress primarily during postnatal development (Barlow et al. 1999; Yan et al. 2001; Yan et al. 2006). Recently, a large-scale proteomic analysis of protein phosphorylation in response to DNA damage revealed that more than 700 proteins and 900 phosphorylation sites were correlated with ATM and ATR (ataxia telangiectasia and Rad3-related) (Matsuoka et al. 2007).

To date, more than 500 *Atm* mutations have been identified as the disease-causing mutations (http://www.hgmd.cf.ac.uk/ac/gene.php?gene=ATM). The mutations can be found in every exon with no apparent hotspots. The majority of *Atm* mutations are frameshift or nonsense mutations (Wright et al. 1996; Concannon and Gatti 1997), which are predicted to truncate the whole ATM protein. Other *Atm* mutations include missense mutation, splicing, and large genomic deletion/duplication, etc.

In China, less than 30 A-T patients have been reported by different hospitals, and only two unique *Atm* mutations have been identified so far (Jiang et al. 2006). This calls a question whether the incidence of A-T in Chinese population is lower than that in other countries or the A-T cases are technically misdiagnosed there. Therefore, it is urgent to study Chinese A-T, including *Atm* mutation analysis. In the present study, we screened 12 novel *Atm* mutations in 8 Chinese A-T patients from 6 unrelated families. Our results showed an inkling that *Atm* mutations in Chinese A-T patients are diverse, which, in turn, make it possible to better identify individual A-T patients who are suitable for future customized mutation-targeted therapies based on their *Atm* mutated status.

## Materials and Methods

### Patients

Eight A-T patients from 6 unrelated families were recruited from 5 different provinces of China. The primary clinical diagnosis for those A-T patients was mainly based on the presence of progressive neurodegeneration as shown by cerebellar ataxia and cerebellar atrophy, telangiectasia, elevated serum levels of alpha-fetoprotein, and altered serum levels of immunoglobulins. The clinical features of the individual A-T patients were summarized in Table 1. All families signed the informed consent for this study.

**Table 1:** Major clinical and laboratory features of Chinese A-T Patients (CHAT)

Patient	Sex	Age	Ataxia-age at onset (month)	Telangiectasia-age at onset (year)	Cerebellar atrophy	Alpha-fetoprotein (ng/ml^)a^	Immunoglobulins			
							IgG	IgA	IgM	IgE
							(g/l)^b^	(g/l)^c^	(g/l)^d^	(KIU/l)^e^
CHAT1	F	13	24	3	Atrophied	554	2.68	0.02	1.1	<0.1
CHAT2	F	4	18	2	Atrophied	142	4.2	0.01	1.5	<0.1
CHAT3	F	14	30	3	Atrophied	651	14.4	0.24	2.47	0
CHAT4	F	7	18	1	Atrophied	164	8.9	0.5	1.26	0
CHAT5	F	13	48	1	Atrophied	325	8.8	1.17	1.07	2.95
CHAT6	M	8	18	2	Atrophied	170	7.8	0.9	1.85	0
CHAT7	M	8	24	2	Atrophied	69	3.5	0.15	1.2	<0.1
CHAT8	F	7	24	2	Atrophied	251	7.72	0.7	0.8	0.12

^a^Range of blood alpha-fetoprotein normal value: 0–20 ng/ml

^b^Range of blood IgG normal value: 7–17 g/l

^c^Range of blood IgA normal value: 0.72–4.29 g/l

^d^Range of blood IgM normal value: 0.6–2.6 g/l

^e^Range of blood IgE normal value: 0–200 KIU/l

### Mutation Screening

Blood samples were collected from each A-T patients and their parents for mutation analysis, which includes the entire *Atm* gene coding sequence, adjacent intron regions and 3′UTR and 5′UTR, and performed by direct sequencing of PCR products as described previously (Soukupova et al. 2011).

The large genomic rearrangements in the *Atm* locus were tested for all patients with the multiplex ligation-dependent probe amplification (MLPA). MLPA is a reliable technology for relatively quantitative analysis of the copy number in clinical diagnosis of genetic diseases. An MLPA kit with probes of P041 and P042 for detecting the deletion and/or duplication of the *Atm* gene was purchased from MRC Holland (Amsterdam, Netherlands). Procedures were performed according to the manufacturer’s instruction. In brief, ligation and amplification were carried out with an ABI 9800 Thermal Cycler. The PCR conditions were 35 cycles at 95 °C for 30 s, 60 °C for 30 s, and 72 °C for 60 s, followed by a final incubation at 72 °C for 20 min. The PCR products were separated by capillary electrophoresis in an ABI 3700 Genetic Analyzer (Applied Biosystems, Foster City, California). The raw data were analyzed by GeneMarker v1.5 software. The peaks obtained after the analysis of DNA fragments could be distinguished and assigned to specific exons on the basis of their different lengths representing the variability of their stuffer sequences. Peak area of raw data was then exported into a Microsoft Excel spreadsheet program to normalize each peak with known normal controls. Peaks derived from A-T patients that vary more than 20 % from the normal controls should be flagged for review. If a deletion of single exon was observed, conventional PCR with primers of the exon was performed to verify the deletion.

## Results

As shown in Table 1, all A-T patients (CHATs) had the typical symptoms of ataxia and telangiectasia, sign of cerebellar atrophy by CT and MRI examinations, and elevated serum alpha-fetoprotein. Immunodeficiency is also one of the major characteristics of A-T. In our Chinese A-T patient cohort, 5 out of 8 patients (62.5 %) had normal or slightly decreased serum levels of IgG, IgA, and IgM. Those configurations were in agreement with the clinical records and patient descriptions that those (CHAT 3, 4, 5, 6 and 8) with relative normal levels of serum immunoglobulins had no histories of repeat sinopulmonary infections, while others (CHAT1, 2 and 7) with agammaglobulinemia often suffered from such infection with fever and had to receive gammaglobulin injection termly. None of the eight A-T patients had sign of being developed a malignancy.

**Fig. 1:**
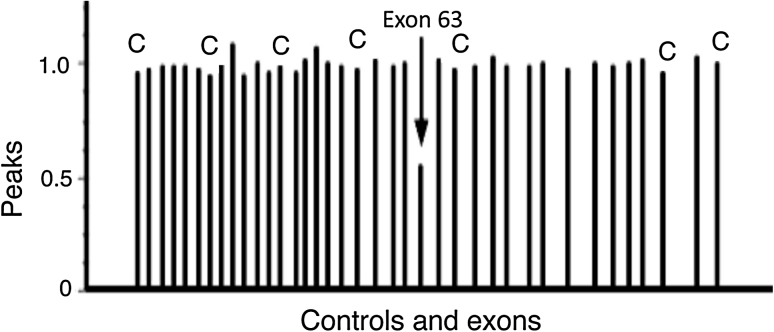
Normalized peak area histogram for LGD analysis in CHAT5. Seven bars with a capital C on the tops represent the signals of control samples, and other 31 *bars* represent the signals of exons detected with the MLPA P041 kit. The *arrow* indicates the decreased signal of exon 63, corresponding to the genomic deletion

The genetic analysis of *Atm* gene identified 12 disease-causing mutations in 6 unrelated A-T families (Table 2). The mutation types are diverse, including 4 nonsense (33.3 %), 1 splicing (8.3 %), 5 frameshift (42 %), 1 large genomic deletion (8.3 %), and 1 missense mutation (8.3). All the *Atm* mutations were novel. Interestingly, a nonsense mutation c.1464G>A in family 1 shared same resultant protein effect of p.W448X with a previously reported nonsense mutation c.1463G>A in an Italian A-T patient (Cavalieri et al. 2006). That is because c.1464G>A results in a premature stop code TGA while c.1463G>A results in a premature stop code TAG. No founder-effect mutation of *Atm* was found because 12 mutations were unique in each unrelated A-T families. In CHAT5, the large genomic deletion in exon 63 was detected in his paternal allele with MPLA method (Fig. 1), combined with a nonsense mutation c.3174G>A in exon 22 in his maternal allele (Table 2).

**Table 2:** *Atm* Mutations of eight patients from six families

Family	Patient	Mutations	Allele	Location	Type	Protein effect
1	CHAT1/2	c.1464G>A	Ma	Exon10	Nonsense	p.W448X
		ins56-1G>A	Pa	Exon57	Splicing	Splicing
2	CHAT3/4	c.2680delG	Ma	Exon18	Frameshift	p.D894IfsX4
		c.7166C>G	Pa	Exon49	Nonsense	p.S2389X
3	CHAT5	c.3174G>A	Ma	Exon22	Nonsense	p.W1058X
		Exon 63 deletion	Pa	Exon63	large genomic deletion	N/A
4	CHAT6	c.2152_2154delinsAAAC	Ma	Exon14	Frameshift	p.C718KfsX19
		c.8713_8714insCA	Pa	Exon60	Frameshift	p.V2906QfsX32
5	CHAT7	c1402_1403delAA	Ma	Exon10	Frameshift	p.K468EfsX17
		c.2413C>T	Pa	Exon16	Nonsense	p.R805X
6	CHAT8	c.6885G>T	Ma	Exon46	Missense	p.V1248F
		c.3742_3743insGGAGGTTCT	Pa	Exon25	Frameshift	p.Y1248WfsX10

## Discussion

Two novel *Atm* mutations in two unrelated Chinese A-T families were reported in 2006 (Jiang et al. 2006). One of them was a homozygous missense mutation, c.1346G>C, and another one was a compound heterozygous nonsense mutation, c.610G>T, combined with a previously reported missense mutation, c.6679C>T. In this study, we screened out 12 novel *Atm* mutations in 6 unrelated Chinese A-T families (Table 2).

The majority of *Atm* mutations are frameshift or nonsense mutations (Wright et al. 1996; Concannon and Gatti 1997; Li and Swift 2000). These mutations result in total loss of ATM protein and account in diagnosis for about 75 % of A-T cases (Jacquemin et al. 2011). Despite the small number of A-T patients we examined, the frequencies of nonsense mutations (4 in 12) and frameshift (5 in 12) are very close to those previously reported (Concannon and gatti 1997).

Large genomic deletion (LGD) is a very rare *Atm* mutation type and had been estimated at 2 % of the *Atm* mutations identified (Cavalieri et al. 2008). However, high frequency of LGD has recently been reported in Japan (Nakamura et al. 2011). Four out of 16 *Atm* mutations in Japanese A-T patients examined were LGDs. There has been no report showing that LGD occurs in a homozygous state in A-T patients (Mitui et al. 2003). In our cases, a LGD in exon 63 was found in CHAT5 whose another mutation was nonsense mutation (c.3174G>A) in exon 22. *Atm* missense mutations have been shown to cause either typical or milder phenotypes (Verhagen et al. 2009). A recent study showed that most *Atm* missense mutations in A-T are functionally associated with expression defects and/or inactivation of ATM protein kinase (Barone et al. 2009). Jacquemin et al. demonstrated that, in addition to causing ATM protein underexpression (15 out of 16 cases), most *Atm* missense mutations exhibited abnormal cytoplasmic localization of ATM (Jacquemin et al. 2011). Among the 12 mutations found in our A-T patients, only one missense mutation in exon 46 combined with a frameshift in exon 25 was identified in CHAT8 whose clinical features of A-T is typical. Together, our results revealed that most *Atm* mutation types, including nonsense, framshift, splicing, missense, and LGD, exist in Chinese A-T patients and that the individual mutations are diverse and distinct largely from those in other ethnic areas.

Although there has been no cure for A-T patients so far, the mutation-targeted therapeutic approaches have recently been developed rapidly, bringing hope of potential treatment for some A-T patients who carry *Atm* mutations suitable for correction by either antisense morpholino oligonucleotide (AMO) or readthrough compound (RTC) (Nakamura et al. 2011). AMOs can effectively correct type II and IV splicing mutations (Eng et al. 2004). Nakamura et al. showed that, using a designed AMO-j11 to treat an A-T cell line, the mutant splicing was abrogated in a dose-dependent manner and the full length ATM protein reappeared in the nuclear extracts from the cells (Nakamura et al. 2011). It has been shown that functional ATM protein can be induced with RTCs towards the premature termination codons in cells with an *Atm* heterozygous nonsense mutation (Du et al. 2009). Using a new designed RTC13, IR-activated ATM autophosphorylation at S1981 was measured in a human A-T lymphoblast cell line (Nakamura et al. 2011). These in vitro studies shed light on clinical application of the customized mutation-targeted therapies for A-T patients in the future. However, this personalized approach basically relies on *Atm* mutation analysis.
